# ERKed by too much signaling: from oncogenic driver to therapeutic vulnerability

**DOI:** 10.1007/s00018-026-06084-6

**Published:** 2026-03-21

**Authors:** Dylan A. Farnsworth, Farhana Naznin, Asha Subramaniam, Katherine Sew, William W. Lockwood

**Affiliations:** 1 Department of Integrative Oncology, BC Cancer Research Institute, 675 West 10th Avenue, Vancouver, V5Z 1L3 BC Canada; 2 Department of Pathology & Laboratory Medicine, University of British, Columbia, Canada

**Keywords:** ERK, Cancer, Hyperactivation, Targeted therapy, MAPK signaling

## Abstract

Extracellular regulated kinase (ERK) signaling is a major driver of cancer development. Mutations accumulated in oncogenes upstream of ERK can promote and sustain tumorigenesis by providing a sustained proliferative signal, helping cells resist death, and inducing angiogenesis. Therapeutic strategies for cancers with dysregulated ERK signaling have focused on inhibiting upstream-mutated oncogenes as a means of depriving tumors of these essential survival cues. While these strategies have demonstrated initial clinical success in patients, they also represent an incomplete understanding of the more nuanced role ERK signaling plays in tumor cell homeostasis. It is now understood that increased ERK signaling can also function as a tumor suppressor by inducing proliferative arrest outside of the framework of oncogene-induced senescence. In this review, we highlight current research describing the vulnerability of cancer cells to ERK hyperactivation induced toxicity and offer insight on how ERK rewiring may be leveraged for the development of new therapeutic strategies for patients.

## Introduction

 The extracellular regulated kinases 1 and 2 (ERK1/2, referred to as ERK) are members of the broader family of mitogen-activated protein kinases (MAPK) and key effectors of the RAS-RAF-MEK-ERK signaling cascade. ERK signaling is initiated by binding of extracellular mitogens, growth factors, cytokines or other signaling molecules to cell surface receptors, triggering a cascade which culminates in ERK phosphorylation and activation. In turn, ERK1/2 can phosphorylate a large set of effector substrates, including other protein kinases, granting it control over key cellular functions including cell survival, proliferation, differentiation and growth [1]. While normal ERK signal transduction is critical for maintaining homeostasis and regulating cell development, dysregulated ERK signaling has important implications in several human diseases, including cancer. Many proteins located upstream of ERK in the ERK/MAPK signaling cascade are mutated in patient tumors, including gain-of-function mutations in isoforms of RAS and RAF that are detected in 19% [2] and 8% [3] of all human cancers, respectively. In an analysis of The Cancer Genome Atlas (TCGA) database, RAS-ERK pathway members were mutated in 46% of tumors, and the pathway bore the highest median mutation frequency across all cancers analyzed [4]. Constitutive activation of the RAS-ERK pathway through gain-of-function mutations accumulated upstream indicates a pro-tumorigenic role for ERK signaling. This role is supported by years of observation of increased ERK signaling across multiple cancer types and association with a multitude of driver oncogenes [5–11], evidence of in vitro transforming potential [12–15], and sensitivity to ERK inhibition across different cancer model systems [16–19]. Taken together, this data has informed drug discovery initiatives and clinical guidelines based on inhibiting malignant ERK signaling driven by mutated oncogenes [20, 21], resulting in increased survival for some genetic subsets of patient [22–26].

While ERK signaling has a well-defined pro-tumorigenic role, there is increasing evidence that it may suppress tumor growth depending on the context. In non-transformed cells, ERK signaling can suppress cell growth by mediating apoptosis and senescence in response to various stimuli [27], including in response to excessive signaling driven by oncogenes [28–30]. Oncogene induced senescence (OIS) represents a powerful tumor suppressive mechanism to protect against hyperproliferation and malignant transformation. However, this process is often mediated by p53 or p16^INK4A^, factors inactivated in many tumor cells, and was found to be dependent on induction of higher than normal levels of sustained ERK signaling [27]. How tumor cells evolve to adapt to high levels of ERK signaling - whether by achieving a balance between pro-growth and pro-death signals, by silencing senescence inducing pathways (i.e. p53 or p16^INK4A^), or through yet undiscovered mechanisms - remains unclear. Recent work has highlighted that cancer cells remain sensitive to excessive signaling through ERK. Several members of the dual specificity phosphatase (DUSP) family – which function as negative regulators of ERK signaling - have been identified as key dependencies in cancer cells [31, 32], highlighting continued need for modulated ERK signaling beyond the stage of initial transformation. Toxicity in response to high levels of ERK has also been observed in across multiple cancer cell models of resistance to targeted therapies in a process referred to as ERK hyperactivation [33–36], raising the prospect of targeting this vulnerability in the clinical setting. As the major node of signaling convergence from multiple commonly mutated oncogenes, inhibition of cell proliferation induced by ERK hyperactivation may also present a shared vulnerability across various cancer types driven by different oncogenes.

In this review, we highlight the current state of knowledge on ERK hyperactivation in the context of cancer biology and therapy. We showcase the cancer types where this phenomenon has been observed, known positive and negative regulators, mechanisms and pathways that mediate loss of proliferative ability, and current efforts to rewire ERK signaling for clinical benefit, with a specific focus on research done in preclinical systems.

## ERK/MAPK signaling

**Fig. 1:**
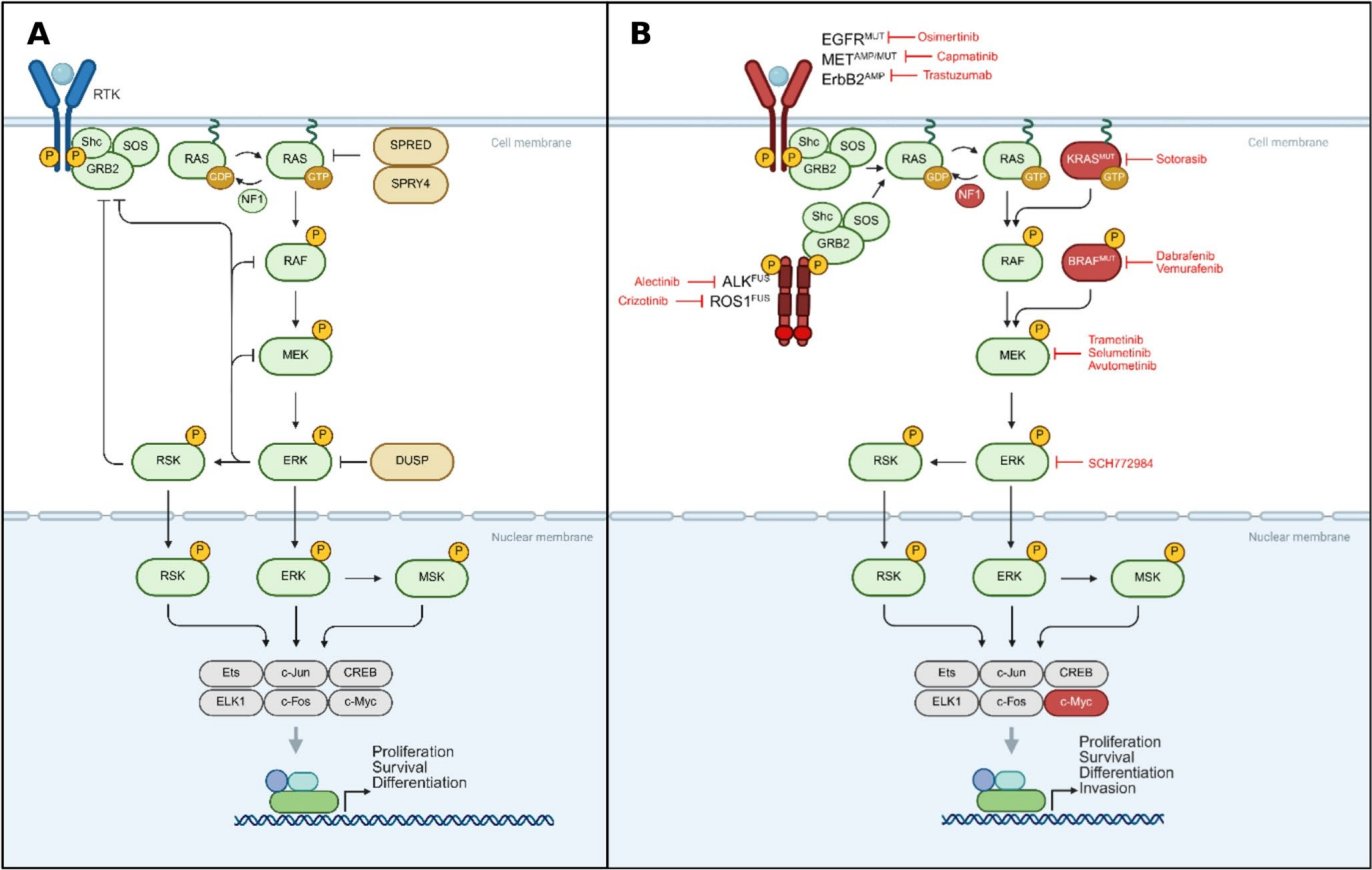
**A** Effectors and regulators of ERK/MAPK signaling in normal cells. **B** Common oncogenic mutations/amplifications affecting the ERK/MAPK signaling pathway and associated targeted inhibitors. Figure made with BioRender

Mammals have four main types of mitogen activate protein kinases (MAPK): ERK1/2, c-Jun N-terminal kinases (JNK1–3), p38, and ERK5. Beyond these primary MAPKs, several atypical MAPKs, such as ERK3/4, ERK7/8, and Nemo-like kinase (NLK), have also been identified. These atypical MAPKs have distinct activation mechanisms and less clearly defined cellular roles [37, 38]. ERK/MAPK signaling is a critical pathway that regulates cellular processes such as development, proliferation, and differentiation [1, 39]. The ERK/MAPK pathway is a highly conserved signaling cascade that transmits extracellular signals to intracellular targets, influencing downstream effects. This pathway consists of a three-tiered kinase signal transduction module: MAPKKK (e.g., RAF), MAPKK (e.g., MEK), MAPK (e.g., ERK1/2) and several MAPKAPKs in the next layer (ribosomal s6 kinases, MAP kinase-interacting serine/threonine-protein kinases, mitogen- and stress-activated protein kinases and cytosolic phospholipase A2) [40, 41] (Fig. 1). Activated ERK translocates to the nucleus, where it phosphorylates hundreds of regulatory molecules and transcription factors to regulate gene expression [39, 42]. MEK1/2 phosphorylates ERK1 at tyrosine residue Tyr204 and threonine residue Thr202, and ERK2 at Tyr187 and Thr185. This dual phosphorylation, which targets both tyrosine and threonine residues within the activation loop of ERK1/2, is a critical regulatory step [41, 43]. ERK1/2-mediated regulatory factors play a crucial role in modulating a range of signaling mechanisms, including the activity of phosphatases that mediate dephosphorylation events (e.g. protein-tyrosine specific phosphatases, protein-serine/threonine phosphatases, and dual specificity phosphatases) [44, 45], the interactions and stability of scaffold proteins that organize and facilitate signaling complexes [46–48], and the regulation of both the duration and intensity of signal transduction processes [49]. Additionally, these regulatory factors govern the dynamic subcellular localization of key components within the signaling cascade, ensuring precise spatial and temporal coordination required for cellular responses [50, 51]. Dysregulation of this pathway is implicated in numerous cancers due to its role in promoting cell survival and cell growth, and thus, oncogenic transformation. Here, we discuss the physiological role of ERK signaling in normal cells, its critical functions in cellular development and proliferation, its pathological activation in cancer cells, and its role in mediating OIS.

### ERK signaling in normal cells

ERK signaling plays a pivotal role in embryogenesis, tissue patterning, cellular proliferation, cell cycle progression, differentiation, survival, metabolism, autophagy and apoptosis [27, 41, 52, 53]. Studies have consistently highlighted its significance across various cellular contexts. During development, ERK coordinates cellular decisions such as proliferation, differentiation, and migration, ensuring proper tissue formation and organogenesis. For example, ERK activation is crucial for correct limb development, where it regulates mesenchymal cell differentiation, and for neural development, where it drives neural progenitor cell maturation and migration [54]. Dysregulation of ERK signaling during development can result in severe congenital abnormalities due to improper cellular communication and impaired tissue organization. In normal cells, ERK signaling regulates cell cycle progression through precise control of cyclin expression and activity. ERK phosphorylates cyclin D1 to promote the G1/S transition. The precise regulation of cyclin expression and activity through ERK signaling highlights its role as a gatekeeper of cell division.

The downstream effects of ERK activation include the initiation of a signaling cascade that impacts chromatin dynamics and gene expression. Activated ERK enhances the activity of its downstream substrates, MSK1 (mitogen- and stress-activated kinase 1) and MSK2 that in turn phosphorylate histone H3 and HMG-14 [3, 23]. These phosphorylation events facilitate chromatin remodeling, allowing transcription factors greater accessibility to target genes. This process plays a crucial role in modulating gene expression in response to ERK pathway activation and is fundamental for various cellular outcomes, such as proliferation, differentiation, and responses to environmental stimuli [55].

Studies have found that ERK dynamics play a critical role in controlling cell fate. In individual cells, stimulation by varying growth factors, oncogenic mutations, and negative feedback structures cause diverse ERK dynamics and different downstream outcomes [56]. At a population level, waves of ERK and AKT signaling are propagated across tissues to maintain cellular homeostasis in response to apoptotic stimuli [57, 58]. ERK is an important regulator of cell survival through both pro- and anti-apoptotic cues. ERK suppresses the pro-apoptotic factor BIM through transcriptional regulation and by stimulating its proteasomal degradation [59, 60], and can also promote expression and stability of anti-apoptotic factors [61, 62]. In contrast, ERK has also been shown to mediate the intrinsic apoptotic response to DNA-damaging agents such as etoposide or radiation, as well as promote cell extrinsic apoptosis by inducing release of death ligands such as TNFα or FasL [27].

ERK signaling also plays a critical role in mediating cellular responses to stress by modulating transcriptional programs and protein stability [63]. These mechanisms maintain genomic integrity in normal cells and ensures the proper functioning of stress response pathways. Furthermore, ERK activation influences the stability and activity of transcription factors involved in DNA repair and apoptosis, thereby protecting cells from the detrimental effects of genomic instability [63, 64]. Additionally, ERK activity can induce permanent cell cycle arrest or trigger autophagic vacuolization, processes often associated with sustained ERK activation localized to specific subcellular compartments [27, 65, 66]. These effects may also depend on the presence of reactive oxygen species (ROS), underlining the complexity of ERK-mediated stress responses. This dual role in promoting both cell survival and repair highlights the adaptive versatility of ERK signaling. By serving as a central regulator of cellular functions, ERK guides critical developmental processes and maintains cellular balance in response to environmental and intrinsic challenges.

### ERK signaling in cancer cells

The ERK/MAPK pathway is indispensable for normal cellular functions but becomes a double-edged sword when dysregulated or hyperactivatedࣧ contributing significantly to the development and progression of cancer [67–69]. The Ras/Raf/MEK/ERK pathway is the most important signaling cascade among all MAPK signal transduction pathways in this context, and plays a crucial role in the survival and development of tumor cells [39, 70]. Aberrant activation of ERK/MAPK signaling is a hallmark of many cancers, including melanoma, lung, ovarian, breast, pancreatic, and colorectal cancers [71–74]. Such overactivation often results from mutations in upstream components like RAS or RAF [72, 75]. Phosphorylated ERK (pERK) is typically activated downstream of receptor tyrosine kinases (RTKs) and RAS proteins via the sequential activation of RAF and MEK kinases [76]. This signaling cascade amplifies external growth signals, enabling cancer cells to evade normal growth control mechanisms. Elevated levels of pERK are associated with enhanced transcription of genes promoting cell cycle progression, metabolic adaptations, and resistance to apoptosis, all of which contribute to tumor progression and metastasis. Elevated levels of pERK drive oncogenic processes by activating transcription factors that regulate genes involved in proliferation and angiogenesis, and enhancing the expression of MMPs (matrix metalloproteinases), facilitating tumor invasion and metastasis [63]. Persistent ERK activation also enables cancer cells to survive under adverse conditions such as hypoxia and nutrient deprivation, further enhancing their adaptability and resilience.

Accumulation of mutations in the MAPK pathway is a common feature in various cancers, further driving its dysregulation (Table 1). Different oncogenic isoforms of EGFR, RAS, RAF, MEK, and ERK vary in their efficacy, function, and, importantly, their potential to cause cancer. Common mutations include EGFR mutations (EGFR^L858R^, EGFR^ex19del^, EGFRvIII), common in lung cancer and glioblastoma [77], RAS mutations (e.g., KRAS^G12C/D/V^, KRAS^G13D^ and KRAS^Q61R^), frequently observed in lung, pancreatic and colorectal cancers [2, 75, 78–80] and BRAF mutations (e.g., V600E), prevalent in melanoma and colorectal cancer [42, 80]. Interestingly, MEK and ERK mutations are less frequently identified in cancers, with MEK mutations reported in approximately 3–8% of melanoma cases and in 3% of colorectal cancer patients [69, 81]. In addition to mutations in RAS and RAF, the loss of negative regulatory mechanisms also contributes to pathway dysregulation. Loss-of-function mutations in NF1, a tumor suppressor gene that encodes a GTPase-activating protein responsible for attenuating RAS activity, lead to sustained activation of the pathway [82]. These mutations often result in constitutive activation of the ERK/MAPK pathway, bypassing normal cellular regulatory mechanisms (Fig. 1b).

**Table 1:** Common MAPK pathway mutations. Data from TCGA

Protein	Lung Cancer	Melanoma	Pancreatic Cancer	Colorectal cancer
*EGFR*	22%	6%	< 1%	2%
*RAS*	26%	26%	76%	46%
*RAF*	7%	36%	2%	14%
*MEK*	1%	7%	< 1%	2%
*ERK*	< 1%	< 1%	< 1%	< 1%

Overall, the ERK/MAPK pathway’s aberrant activation underpins the aggressive nature of many cancers. Its role in driving tumor proliferation, invasion, metastasis, and survival underlines its potential as a therapeutic target. Pharmaceutical efforts have yielded therapies targeting either mutated oncogenes or important signaling nodes (Fig. 1b). However, the pathway’s complexity and potential for adaptive resistance to targeted agents underscore the need for combination therapies to achieve sustained therapeutic efficacy [70, 83]. Understanding the molecular basis of ERK/MAPK pathway dysregulation and its downstream effects remains a critical area of focus in cancer research, with significant implications for the development of targeted therapies.

### Oncogene induced senescence

The RTK-RAS-RAF-MEK-ERK signaling pathway is oncogenically activated in approximately 25% of all human cancers. However, excessive activation of RAS-BRAF-MEK-ERK pathway triggers a sustained and largely irreversible cell-cycle arrest in non-transformed cells known as oncogene-induced senescence (OIS) [84, 85]. OIS is a cellular response that functions as an intrinsic tumor-suppressive mechanism, acting as a barrier to tumor development and progression [86–88]. The process is primarily mediated through a sustained DNA damage response (DDR), upregulation of p53/p21^Cip1^ and p16/RB signaling pathways, the formation of senescence-associated heterochromatic foci (SAHF), increased activity of senescence-associated beta-galactosidase (SA-ßGal), and the presence of a senescence-associated secretory phenotype (SASP), a complex network of cytokines, chemokines, and growth factors and the induction of cell cycle arrest [84, 89–91]. Despite its tumor-suppressive role, additional genetic and epigenetic alterations can override OIS, facilitating cancer progression [92–94].

Interestingly, ERK hyperactivation can lead to two contrasting cellular outcomes: uncontrolled proliferation or senescence, depending on the cellular context and duration of activation. Understanding the interplay between OIS and ERK activation is critical for deciphering the fine balance between senescence as a tumor-suppressive mechanism and its potential to drive tumor progression. Increased activation of the ERK pathway is a hallmark of many cancers and is often driven by oncogenic mutations in upstream regulators such as KRAS (e.g., KRAS^G12D^), BRAF (e.g., BRAF^V600E^), and MEK, resulting in constitutive pathway activation [84]. While transient ERK activation promotes cellular proliferation and survival, sustained ERK activation induces cellular stress, including replicative stress, oxidative damage, and DNA damage, all of which are defining characteristic of OIS, primarily mediated through the upregulation of tumor suppressor proteins such as p16^INK4A^/Rb and p53/p21^Cip1^ [1, 78, 95–97]. Prolonged expression of oncogenic Ras leads to permanent cell-cycle arrest in primary human and rodent fibroblasts through the constitutive activation of MEK [28, 84]. MEK overexpression drives excessive replication stress, causing senescence through activation of the p53/p21^Cip1^ axis, while mutant BRAF^V600E^ induces OIS in melanoma and other cancers via sustained MAPK pathway activation [3, 4, 28, 89]. In fibroblasts expressing oncogenic RAS, sustained ERK signaling lead to the induction and maintenance of the senescent phenotype [98].

ERK directly and indirectly influences the transcription of key genes involved in senescence, reinforcing cell cycle arrest and senescence maintenance. Although sustained ERK signaling can enforce OIS, cancer cells often acquire mutations or undergo epigenetic changes that allow them to bypass this senescent barrier. Loss-of-function of p53 or p16^INK4a^ or mutations in these tumor suppressors are the most common mechanisms that enable cancer cells to evade OIS and resume proliferation in response to ERK [92–94, 99]. ERK signaling often triggers negative feedback loops, including the activation of dual-specificity phosphatases (DUSPs), which dampen sustained ERK activity, allowing cells to bypass senescence [31, 32]. Additionally, crosstalk between ERK and PI3K-AKT signaling pathways can alter cellular outcomes, allowing escape from senescence [100]. The paradoxical role of SASP in cancer progression lies in its ability to both suppress and promote tumorigenesis. While it acts as a powerful barrier against early tumorigenesis, prolonged SASP exposure creates a pro-inflammatory tumor microenvironment, supporting tumor progression [101]. Ultimately, the dual role of OIS in both preventing and facilitating tumor progression highlights its significance in cancer biology [89].

### ERK hyperactivation in cancer models: evidence of toxicity

While ERK signaling is understood to mediate toxic effects to normal cells through activation of OIS, research has revealed that excessive ERK signaling is also be toxic to cancer cells. The mechanisms and triggers regulating ERK toxicity to cancer cells, as well as potential therapeutic strategies, are active areas of research. Much of the early evidence for the potential of ERK signaling to be detrimental to cancer cells comes from studies with similar design to those that discovered OIS. Researchers overexpressed common oncogenes in cancer models and observed similar growth inhibitory effects as in normal cells.

#### Overexpression of ERK regulators and induction of toxicity in cancer cells

In three separate studies, Ravi and colleagues studied the effect of MAPK activating mutations in models of small cell lung cancer [102, 103] and prostate cancer [104] using a fusion protein composed of the kinase domain of CRAF (Raf1) and the hormone binding domain of the human estrogen receptor. In this construct, the truncated form of CRAF is under the control of estradiol, and was previously shown to activate ERK/MAPK signaling [105, 106]. In H209 and H510 cells, activation of ΔRaf-1:ER led to increased ERK signaling, as measured by ERK phosphorylation, decreased colony forming potential, and induction of the cyclin dependent kinase inhibitor (CDKI) p27^Kip1^. They also found they could partially rescue induction of p27^Kip1^ by treating with a MEK inhibitor, implicating ERK as a mediator of reduced fitness [102]. In a separate model of SCLC, DMS53, they found activation of ΔRaf-1:ER led to induction of the CDKI p16^INK4A^, along with cell cycle arrest and decreased colony forming potential [103]. In prostate cancer, they again observed cell cycle arrest and decreased colony-forming ability upon induction of ΔRaf-1:ER. Here, however, they observed upregulation of the CDKI p21^Cip1^ [104]. With these studies, the authors demonstrate a shared mechanisms of proliferative arrest driven by CRAF: cell cycle arrest following induction of CDKIs. However, the specific CDKI mediating the response was cell type specific.

In a 2001 study, Fanton and colleagues induced ΔRaf-1:ER in astrocytic cells and high-grade glioma cells [107]. In astrocytes, CRAF signaling drove irreversible growth arrest accompanied by high levels of p16 ^INK4A^ expression. In glioma models that lack p16^INK4A^/pRb signaling, ΔRaf-1:ER activation led to reversible growth arrest accompanied by increased p21^Cip1^ expression. This work also highlighted cell specific responses to increased CRAF signaling. While the authors observe increased phosphorylated ERK signaling, they did not directly show dependence on ERK signaling mediating the loss of proliferation [107].

In a 2003 study, Park and colleagues investigated the mechanisms by which CRAF overexpression induced proliferative arrest [108]. In the medullary thyroid cancer cell line TT, they overexpressed CRAF using a similar construct as those used for earlier studies. They also found that it induced ERK mediated cell cycle arrest upon overexpression of ΔRaf-1:ER. They next investigated whether cell extrinsic signaling might drive loss of proliferation. They isolated leukemia inhibitory factor (LIF), a cytokine, from conditioned media, and found that it could recapitulate the growth inhibitory effects of ΔRaf-1:ER induction. Further investigation revealed that LIF induced growth arrest through JAK/STAT3 signaling activation, which could be rescued with an inhibitor for the pathway. Revisiting previously generated models, they noted overexpression of CRAF in the SCLC lines H209 and H510 also induced LIF. While LIF alone could drive proliferative arrest by paracrine and autocrine activation of STAT3, they also found that cell intrinsic ERK signaling induced by CRAF was also toxic to cells through an independent, uncharacterized mechanism [108].

Hong and colleagues overexpressed ΔRaf-1:ER in prostate cancer cell line LNCaP. They found that ΔRaf-1:ER activates ERK signaling, induced morphological changes and decreased cell proliferation accompanied by p21^Cip1^ upregulation [109], similar to what was previously observed [104]. They were able to rescue the loss of proliferation by genetically suppressing both ERK1 and ERK2 together, but not individually, suggesting a redundant effect of the isoforms in mediating proliferative arrest. The authors next investigated the role of ERK activation by re-expressing ERK2 bearing mutations either in the kinase domain or in the activation loop, in the background of ERK1/2 knockdown. In this model system, they found both the kinase dead and the activation loop mutant could partially induce the growth inhibitory phenotype. This suggests that both catalytic and non-catalytic ERK signaling can mediate proliferative arrest [109]. Follow up revealed that the growth inhibitory phenotype and induction of CDKIs was driven by ERK dependent suppression of androgen receptor (AR) signaling, important for survival of prostate cancer cells [110]. Guegan and colleagues made similar observations of ERK1/2 redundancy in models of breast and liver cancer [111]. Using a constitutively active MEK mutant, they found that both ERK1 and ERK2 could mediate proliferative arrest and upregulate p21^Cip1^, although they note better rescue of the phenotype upon ERK2 deletion. They also observed that proliferative arrest was mediated by kinase dependent and independent ERK signaling. Next, they partially rescued both p21^Cip1^ induction and proliferative arrest by inhibiting p70S6K, implicating it as a mediator of toxicity downstream of ERK [111].

Building their previous work with ΔRaf-1:ER overexpression, Hong et al. identified a threshold for ERK activity that underlines a switch from a growth arrest to cell death. They overexpressed wild-type ERK1 and ERK2 individually in their previously established ΔRaf-1:ER overexpression LNCaP cell line [112]. Instead of cell cycle arrest and induction of CDKIs, they observed caspase dependent cell death, again mediated by ERK. They next attempted to rescue this phenotype by titrating with a MEK inhibitor. By titrating a MEK inhibitor, they found a threshold for ERK suppression at which the cells switched from cell death back to cell cycle arrest. This indicates that the type of proliferative arrest induced by hyperactive ERK is itself dependent on ERK levels. Extreme levels of ERK led to apoptosis, whereas high levels led to cell cycle arrest. Further work revealed that ERK kinase activity was necessary to mediate cell death [112], where they showed that cell cycle arrest was both ERK kinase dependent and independent [109].

Drosten and colleagues sought to assess the bioequivalence of KRAS and HRAS mutations by overexpressing HRAS^G12V^ or KRAS^G12V^ from the KRAS coding locus in adult mice [113]. While KRAS^G12V^ expression led to adenocarcinoma lesions, HRAS^G12V^ expression led to no lung tumor development, but instead induction of senescence markers, despite increased GTP-bound RAS and increased pERK staining. Ablation of p53 or co-treatment with the MEK inhibitor trametinib allowed for the survival of HRAS^G12V^ expressing cells, indicating that excessive ERK signaling and p53 induction were toxic to lung cells. The authors performed similar experiments in cells from other tissues. Global expression of either KRAS^G12V^ or HRAS^G12V^ led to different patterns of malignancies, including more papilloma and hematopoietic malignancies in HRAS^G12V^ expressing mice, and more lesions with KRAS^G12V^ when expressed in acinar cells. While the authors did not further characterize the role of ERK signaling in these contexts, this study hints at the different capacities for spare ERK signaling across different tissue types.

- Caesar and colleagues aimed to investigate the rationale for ERK signaling suppression in SCLC with the updated framework of SCLC subtypes. They overexpressed a constitutive active form of MEK1 in cell models from the 3 predominant subtypes of SCLC (ASCL1-high, NEUROD1-high, POU2F3-high) [114]. In the ASLC1-high subtype, they found that MEK1 signaling induced G2 cell cycle arrest and senescence. They also observed STAT3 activation, consistent with previous results [108], however did not investigate if STAT3 activation was mediated by LIF, as previously reported [108]. Work from our group also found ERK signaling induced by KRAS^G12V^ signaling induced proliferative arrest in the ASCL1-high cell line H2107, although was also detrimental in the NEUROD1-high line H524 [115].

#### Direct ERK overexpression mediated effects

Since the discovery that increased ERK signaling can be toxic to cancer cells, several groups have aimed to model this phenotype through direct overexpression of ERK. Wu and colleagues demonstrated that overexpression of the constitutively active ERK2^L73P/S151D^ mutation was sufficient to induce proliferative arrest and upregulation of p21^Cip1^ and p16^INK4A^ in LNCaP prostate cancer cells [116], although the effects were not as strong as with constitutively active MEK1 mutant characterized in previous work [117]. Goetz and colleagues screened for ERK1 and ERK2 mutations that could induce resistance to ERK pathway inhibitors in A375 melanoma cells. Although their study was mainly focused on this angle, they also identified ERK1 and ERK2 mutations that triggered loss of viability when expressed in the absence of drug [118]. Brenan and colleagues aimed to functionally characterize ERK2 variants in the context of ERK inhibitors. They generated a cDNA expression library composed of all possible amino acid substitutions at residues 2–360 of ERK2, then overexpressed the library in A375 cells [119]. They observed depletion of several known ERK2 activating mutants in their screen, including the previously characterized ERK2 seven maker mutation [119]. Here again, the study was focused on characterizing which domains may be important for resistance to ERK inhibitors, and as such did not further characterize mutations that induced loss of viability when expressed in the absence of drug.

In a 2019 study, Leung and colleagues used a doxycycline (dox) inducible construct to overexpress wild-type ERK in BRAF mutant melanoma cell lines [120]. They found that overexpression of either wild-type ERK1 or ERK2 was sufficient to mediate proliferative arrest. They also investigated whether cell extrinsic signaling factors may drive loss of proliferation. Conditioned media from cells overexpressing ERK2 induced loss of proliferation in parental cells, although not as strongly as ERK2 overexpression, suggesting cell extrinsic signaling mediated a portion of the anti-proliferative effects. The authors next analyzed secreted factors by proteomics and reported upregulation of LIF upon overexpression of ERK, consistent with previous reports [108], but did not follow up further. The authors observed cell death and activation of apoptotic caspases upon overexpression of ERK2, however found that cell death was caspase independent. They noted that cell death from ERK2 overexpression only occurred in cells with RAF or RAS mutations, findings later recapitulated in an open reading frame screen on MAPK pathway activation [121]. This was consistent with their ability to rescue proliferative arrest by treating with ERK pathway inhibitors, and suggests that overexpressed ERK was still dependent on upstream oncogenes for activation beyond a tolerable level [120].

Soudah and colleagues generated a genetically engineered mouse model to study the ERK signaling in hepatocellular carcinoma. They expressed the constitutively active ERK1^R84H^ in mouse livers [122]. They found that while ERK1^R84H^ expression in the liver did induce hepatocellular carcinoma, it also resulted in decreased phosphorylation of ERK in the tumors. This was mirrored in NIH3T3 cells, where ERK1^R84H^ induced transformation but also resulted in decreased ERK signaling. While not directly demonstrating deleterious effects of ERK, the authors did find that a strong negative feedback loop was necessary for transformation induced by ERK1^R84H^.

### Toxicity mediated by inhibition of negative regulators

The use of inducible oncogenes in in vitro cancer models presents useful systems to study the growth dynamics and cell signaling associated with ERK hyperactivation. However, most models rely on overexpressing oncogenes to super-physiological levels and kill cancer cells in ways challenging to reproduce in a clinical setting. If this vulnerability is to be exploited in patients, other systems and targets need to be studied. Several groups have investigated targeting negative regulators of ERK/MAPK signaling as a means of inducing ERK hyperactivation.

Dual-specificity mitogen activated protein kinase phosphatases (MKPs or DUSPs) represent a common class of ERK/MAPK signaling regulators that dephosphorylate tyrosine and threonine residues within the activation loop of mammalian MAPKs (ERK, p38, JNK) [123]. In cancer contexts, individual DUSPs have been observed as both tumor suppressive and pro-oncogenic [123]. Rahmouni and colleagues deleted VHR (DUSP3), a dual specificity phosphatase, and found it induced cell cycle arrest, upregulation of p21^Cip1^, and senescence in HeLa cells. This phenotype could be partially rescued through inhibition of MEK signaling, although the authors found greater rescue when also knocking down JNK, suggesting multiple hyperactivated pathways were responsible for the phenotype observed [124].

In a follow up to their previous study, Wu and colleagues aimed to identify key regulators of proliferative arrest induced by hyperactive ERK [117]. In LNCaP cells, they induced hyperactive ERK by overexpressing a constitutively active version of MEK1 that was also HA-tagged for tandem affinity purification. Using this system, they isolated mortalin (HSPA9), an Hsp70 family protein, as a binding partner of constitutively active MEK1. They found that mortalin acted to negatively regulate ERK signaling, protecting cancer cells from p21^Cip1^ and p16^INK4A^ expression. In BRAF or KRAS mutant cell lines, mortalin depletion led to upregulation of ERK signaling, ERK dependent cell cycle arrest through p53-dependent and p53-independent mechanisms, and caspase independent cell death, although the response varied by model [117]. In later work, Karkhanis and Park elucidated mechanism by which ERK activates Sp1, a transcription factor, which in turn activated p21^Cip1^ transcription, demonstrating p21^Cip1^ induction independent from p53 activity [125].

A mechanism by which mortalin, a chaperone protein, might regulate ERK MAPK signaling came in a later study published in 2017. In cell models of melanoma and pancreatic cancer, they found that mortalin facilitates direct interaction between MEK1 and PP1A, a phosphatase that suppresses ERK signaling by dephosphorylating MEK [126]. In later studies, mortalin was observed to protect BRAF mutant melanoma cells ^*1–3*^, KRAS mutant pancreatic ductal adenocarcinoma (PDAC), and colorectal cancer (CRC) cells [127] from mitochondrial permeability dysregulation. Mortalin was found to directly interact with components in the mitochondrial membrane. Its deletion was highly lethal in cancer cells with dysregulated ERK signaling, highlighting its therapeutic potential in these settings [127–129].

Ingram and colleagues investigated the relationship between increased pERK signaling and de-differentiation of lung adenocarcinoma (LUAD) cells [130]. They found that the lineage transcription factor NKX2-1/TTF1 induced DUSP6 expression. In mouse and cell models, they demonstrated that NKX2-1 overexpression led to decreased cancer cell proliferation through upregulation of DUSP6, suggesting NKX2-1 slows tumor progression by limiting ERK signaling. However DUSP6 knockout was also found to decrease cell proliferation and migration through ERK hyperactivation [130]. Their work indicates that cells select for optimal NKX2-1 expression for its modulation of ERK signaling, mediated by DUSP6. In early tumors, DUSP6 downregulation promotes tumor development, however in later tumors it is important to maintain regulation of ERK signaling [130].

Guttierez-Prat and colleagues inhibited DUSP4 in NRAS and BRAF mutant melanoma. They found that DUSP4 deletion resulted in ERK dependent proliferation decrease and modest induction of apoptosis [131]. Additionally, they observed a decrease in the expression of the melanocyte lineage transcription factor MITF upon hyperactivation of ERK by DUSP4 deletion, recapitulating findings from models of BRAF inhibitor addiction [34]. Valcikova and colleagues aimed to investigate the interplay between ERK signaling and the translation initiation factor eIF4F [132], which has been associated with resistance to BRAF and MEK inhibitors in melanoma [133]. Mass spectrometry revealed that eIF4F inhibitor treatment resulted in increased ERK signaling, with no changes to MEK or RAF activation. They observed rapid decrease in levels of DUSP4, DUSP6 and DUSP7 proteins, but also an increase in DUSP4 and DUSP6 transcript levels, suggesting post-translational regulation. While the authors did not characterize effects of eIF4F inhibition on cell dynamics in this study, eIF4F inhibition has been preciously shown to decrease melanoma persister cells when combined with MEK and RAF inhibitors [134].

### Synthetic lethality of oncogenic mutations

More evidence for the potentially deleterious effects of ERK signaling in cancer cells comes in the form of mutual exclusivity patterns between mutated oncogenes with similar signaling functions. Mutations in *EGFR*, *RAS* or *BRAF*, all key effectors of ERK/MAPK signaling, usually occur in a mutually exclusive manner across multiple cancer types [135–141], although can co-occur in tissue or treatment specific instances [142]. Mutual exclusivity has been hypothesized to result from functional redundancy in genes. Many mutually exclusive oncogenes signal through the same pathway, where a second activating mutation to a separate effector might provide no selective advantage. Another explanation is that mutually exclusive mutations are synthetically lethal and induce negative selection pressure in cells where they occur. While statistical methods applied to patient sequencing data can determine the odds that mutually exclusive patterns occur by chance [141], in vitro or in vivo testing is required to validate the effect of oncogenes on cells dynamics.

In 2015, Unni et al. analyzed the mutual exclusivity between constitutively activating EGFR and KRAS mutants in LUAD, and found that mutual exclusivity was driven by synthetic lethality, not functional redundancy of oncogenes [139]. Co-expression of mutant KRAS and mutant EGFR in mouse lungs resulted in tumors expressing only one of the two oncogenes, suggesting strong negative selection pressure against cells harboring both oncogenes. Co-expression mutant KRAS and mutant EGFR in cell models of LUAD resulted in increased ERK, AKT and p38 signaling, increased vacuolization, macropinocytosis, and induction of both apoptosis and methusosis. Ambrogio and colleagues found similar results murine models of LUAD, where coexpression of EGFR^L858R^ and KRAS^G12V^ induced replicative stress and apoptosis in established tumors [143]. In a follow up study three years later, Unni and colleagues identified ERK2 as the key mediator of toxicity induced by coexpression of mutant EGFR and mutant KRAS, confirming ERK hyperactivation as the selection pressure driving mutual exclusivity of these oncogenes [32]. They also identified DUSP6 as the most significantly upregulated ERK suppressor in LUAD patients with either KRAS or EGFR mutations relative to patients not bearing these mutations. Pharmacological or genetic suppression of DUSP6 resulted ERK hyperactivation and loss of viability in LUAD models, similar to that observed upon co-expression of mutant EGFR and mutant KRAS [32].

Cisowski and colleagues performed similar experiments in a 2016 study aimed at understanding the mutual exclusivity of mutant BRAF and KRAS. They co-expressed constitutively active BRAF^V600E^ and KRAS^G12D^ in mouse lungs, and found mice expressing both oncogenes had a lower tumor burden relative to those with individual oncogenes [144]. IHC on tumors found that higher levels of ERK and AKT signaling were associated with lower levels of cell proliferation markers. Authors also found elevated levels of cell cycle inhibitory genes from the *Ink4a/Arf* locus in cells co-expressing both oncogenes, reminiscent of oncogene induced senescence. The authors did not observe induction of apoptosis or increases in negative ERK regulators *Dusp2*, *Dusp4*, *Sprouty2*, *Sprouty4* and *Spread4*. Observations of senescence markers mirror previous work in melanoma, where overexpression of constitutively active NRAS^Q61R^ in a BRAF^V600E^ mutant melanoma clone increased ERK and AKT signaling and induction of senescence after several passages [145]. In this study, the authors also found that cells expressing both oncogenes were more susceptible to cell mediated toxicity, suggesting an immune component to the negative pressure exerted by BRAF and NRAS co-expression [145].

Chang and colleagues took a more systematic approach to query sensitivity to signaling hyperactivation. They performed an open reading frame, gain of function screen across 488 barcoded cell lines, using activators from the PI3K/AKT, ERK/MAPK and Wnt pathways. They found that cells bearing RAF or RAS mutations were sensitive to further MAPK activation, whereas cells with no MAPK activating mutation or receptor tyrosine kinase activating mutations were not. Through a 214 antibody panel applied to over 400 cells lines, they demonstrated that cells with higher levels of MEK phosphorylation had higher sensitivity to MAPK activating mutations, validating high ERK signaling as a sensitizer for MAPK mutation induced lethality. They also showed for the first time sensitivity to PI3K pathway activation in endometrial cancer and sensitivity to Wnt pathway activation in Wnt driven cancers, highlighting sensitivity to hyperactivation of other important cancer pathways in context dependent settings [121].

### Mechanism of ERK mediated toxicity uncovered through functional genomic screens

Large scale genomic alteration screens have proven to be some of the most effective tools to uncover novel vulnerabilities in specific cancer subtypes. Christodoulou and colleagues aimed to uncover melanoma specific gene dependencies by probing previously published CRISPR-Cas9 dropout screen results. They first identified melanoma specific dependencies from screens in 28 cell lines, then cross referenced results against data from 313 cell lines representing 18 other tumor types [146]. From this analysis, they uncovered multiple negative mediators of ERK/MAPK signaling, including DUSP4 and PPP2R2A, as dependencies in melanoma. These two were validated in separate melanoma models and found to decrease cell proliferation when genetically suppressed, however no further profiling was performed [146]. Kamada and colleagues took a similar approach by probing the gene-dependency database DepMap [147]. They also found that melanoma cells, relative to cell lines of other cancer types, were more dependent on DUSP4 for survival [148]. In their melanoma models, they observed that DUSP4 suppression resulted in decreased cell proliferation, however, was also associated with a decrease in pERK. Further investigation revealed that this was due to a compensatory effect of DUSP6, which is post-translationally upregulated following DUSP4 suppression [148].

Cho and colleagues aimed to screen for genes mediating sensitivity to MEK inhibitors in melanoma. They performed a shRNA screen with a custom library targeting genes encoding kinases or phosphatases on the melanoma cell line 501 grown in different concentrations of a MEK inhibitor [149]. They uncovered PPP6C, a phosphatase that regulates ERK signaling by acting on MEK, as a key mediator or MEK inhibitor response. Under conditions of trametinib treatment, loss of PPP6C promoted phosphorylation of MEK, which in turn induced ERK signaling and resistance to MEK inhibitors. This same mechanism resulted in PPP6C loss being deleterious to cells when grown in the absence of trametinib, likely due to ERK hyperactivation. The authors do not further characterize the proliferative arrest driven by PPP6C loss.

Ito and colleagues developed a novel method to screen genes with functional paralogs, a class of targets usually missed in traditional single gene perturbation screens. They generated a guide library targeting genes from the hydrolase, ligase and transferase protein classes, and tested it across cancer cell lines from multiple origins [31]. They identify DUSP4/DUSP6, a pair of phosphatases, as being lethal when deleted in NRAS or BRAF mutant melanoma cells due to their suppression of ERK. Interestingly, this effect was only true in cells with mutations to NRAS or BRAF and was not present in NRAS^WT^ cells. Additionally, they found that the anti-proliferative effects of DUSP4/6 inhibition are more potent in a melanoma model resistant to MAPK-pathway inhibitors. The drug resistant cell line they assessed had elevated levels of ERK signaling relative to its parental counterpart. The authors propose that cells resistant to MAPK pathway inhibitors through alterations that increase ERK signaling may be further sensitized to DUSP4/inhibition [31].

### Drug addicted cell models

Some of the most enticing evidence for the translational potential of the ERK hyperactivation phenotype comes from the study of “drug addicted” cells. Cancer cells bearing actionable oncogenic mutations, treated continuously with targeted therapies, eventually develop resistance to these therapies. In some cases, the mechanism of resistance is only tolerated in the presence of the drug, which buffers oncogenic signaling to a non-toxic level. Drug removal initiates the signaling cascade and induces tumor regression without necessitating secondary interventions. This phenotype has been characterized across a variety of cancer types, primarily following long-term treatment with ERK/MAPK inhibitors. This data suggests a treatment strategy based on intermittent dosing aimed at harnessing signaling instability of drug resistant cells.

The only clinical evidence of drug addiction and potential benefits of intermittent dosing to forestall drug resistance comes from treatment of melanoma patients with BRAF^V600E^ inhibitors. In 2012, clinicians reported on 2 melanoma patients who were successfully re-challenged with a selective BRAF inhibitor after initial treatment, progression and discontinuation of treatment [150]. A mechanism of resistance to the BRAF inhibitors was not reported, and patients received treatment with ipilimumab in the interim time between BRAF inhibitor treatments. Other reports of successful re-challenge with BRAF inhibitors followed [68, 151–155], however mechanisms of resistance to initial rounds of treatments with BRAF inhibitors were not reported.

A set of in vitro and in vivo studies provided some biological rationale for these successful re-challenges. Das Thakur and colleagues used a patient derived xenograft (PDX) melanoma model to profile resistance to vemurafenib [33]. One resistant tumor was found to have elevated levels of ERK signaling relative to the drug naïve cells, and have higher levels of BRAF transcripts, in line with previous reports of vemurafenib resistance [156]. These tumors demonstrated bell-shaped response curve to both vemurafenib and a MEK inhibitor, highlighting sensitivity in these cells to both too low levels and too high levels of signaling through the ERK/MAPK pathway. They re-implanted these cells into mice and leveraged an intermittent dosing schedule to delay emergence of resistant clones and increase survival [33]. Another study by Sun and colleagues highlighted how EGFR upregulation, a separate mechanism of resistance to vemurafenib [157], was also only tolerated in the presence of vemurafenib [158]. They found that overexpression of EGFR in BRAF mutant melanoma models was deleterious in the absence of RAF or MEK inhibitors, leading to upregulation of markers of senescence, but beneficial to cells in presence of these inhibitors [158]. Together, these studies provide distinct molecular basis on how resistance to BRAF inhibitors may emerge at the cost of fitness in the absence of drug.

Subsequent studies exploring addiction to BRAF or MEK inhibitors in melanoma models have explored the pathways mediating loss of fitness and corresponding mechanism of cell death. Moriceau and colleagues studied the mechanisms of resistance to BRAF and MEK inhibitor combination therapy in melanoma, a clinical regimen found to be more effective than BRAF inhibitor alone [159]. They identified *BRAF*^*V600E*^ ultra-amplification (> 160 copies) and *BRAF*^*V600E*^ amplification (20 copies) with *MEK1*^*F129L*^ mutations as resistance mechanisms reactivating ERK signaling while also leading to drug addiction. They demonstrated rescue of the drug addiction phenotype with an ERK inhibitor and showed that the fold increase in pERK rebound upon drug removal was correlated with the loss of viability observed, highlighting ERK signaling as the mediator of decreased proliferation. Of note, they found that double drug resistant cells have higher pERK rebound and proliferation inhibition relative to the single drug resistant cells. The authors also probed patient radiologic data, but generally found progression after cessation of BRAFi + MEKi treatment, despite some cases of tumor regression [159].

A follow up study from the same group three years later investigated the kinetics of MAPK reactivation in drug resistant models. Using similar single and double drug resistant lines, they defined two distinct responses to drug removal: a transient cell cycle slow down and a caspase independent, parthanatos related, cell death response, that were dependent on pERK rebound levels [160]. Researchers also sought strategies to augment the effect of pERK rebound on cells. Given the observation of DNA damage following drug removal and pERK rebound, they were able to potentiate drug addiction by inhibiting DNA damage response. In cells with atypical BRAF mutations or NRAS mutations, they were able to potentiate drug addiction through paradoxical activation of ERK signaling using vemurafenib. Given their previous observations of limited effects of drug removal on patients, the authors argue that successful leveraging of the drug addiction phenotype may require additional interventions [160].

Kong and colleagues aimed to identify the mediators of toxicity downstream of ERK in models of drug addiction in a 2017 study. To this end, they generated dabrafenib resistant and addicted cells, then performed a genome wide CRISPR-Cas9 knockout (KO) screen. Across multiple models of drug addiction, they identified ERK2, JUNB and FRA1 as key mediators of the drug addiction phenotype [34]. The functional distinction between ERK1 and ERK2 was highlighted again in this study, as ERK1 depletion had no effect on the drug addiction phenotype, whereas ERK2 depletion rescues loss of viability induced by drug removal. Downstream of ERK2, they identified JUNB and FRA1 as key transcription factors (TF) inducing downregulation of MITF, a key TF mediating melanocyte lineage survival. Additionally, they noted ERK2 driving a concurrent switch to a more invasive EMT like phenotype and promoting massive cell death, which was enhanced by treatment with dacarbazine, although they do not investigate which kind of cell death [34].

Wang and colleagues sought to profile fitness costs associated with the drug resistant state in melanoma [161]. In A375 cells, they generated populations resistant to either single drug (vemurafenib) or double drug (dabrafenib and trametinib), and observed modest sensitivity to drug removal, consistent with previous work [34, 159, 160]. They found that the drug resistant state was associated with increased sensitivity to ROS induced by the histone deacetylase inhibitor vorinostat. Treatment with vorinostat had antitumor effects in both cell and mouse models of drug resistance. Cycling of vorinostat and MAPK pathway targeted therapy was evaluated in a clinical trial (NCT02836548); however durable response was only observed in 9% of patients [162].

While most of the direct evidence of drug addiction comes from the study of BRAF inhibitor resistance, it has been observed in other cancer types. Preceding reports in melanoma, Suda and colleagues observed drug addiction in a LUAD model in 2012. They treated HCC827 cells with CL-387,785, an irreversible EGFR inhibitor, and reported resistance through loss of PTEN and upregulation of EGFR [163]. They found this line to be dependent on continued culture in CL-387,785 or the MEK inhibitor selumetinib for survival [163]. This specific cell line was later reanalyzed by Kong and colleagues, and also found to be dependent on suppression of ERK2 for survival in the absence of drug [34]. Work from our own group has uncovered KRAS^G12C^ amplification as a mechanism of acquired resistance to trametinib, a MEK inhibitor, that also resulted in drug addiction in a model of LUAD [36]. We treated H358 cells continuously with trametinib until they became resistant and observed dependence on culture with trametinib for survival in one cell line. We were able to recapitulate previously observed functional distinctions between ERK1 and ERK2, while also observing upregulation of markers of endoplasmic reticulum (ER) stress, markers of EMT, and induction of apoptosis upon trametinib removal. Rescue of apoptosis and proliferative arrest with the KRAS^G12C^ inhibitor sotorasib validated amplified KRAS^G12C^ as the driver of ERK hyperactivation in the absence of drug [36].

In models of colorectal cancer (CRC), Sale and colleagues studied resistance to MEK inhibitors. They recapitulated previously observed BRAF^V600E^ amplification and KRAS^G13D^ mutations as mechanisms of resistance to selumetinib. In the BRAF^V600E^ amplification line, they demonstrated cell cycle arrest and induction of apoptosis following ERK hyperactivation induced by drug removal, resulting in a reversible drug resistant state [35]. In the cells resistant through KRAS^G13D^ mutation, they reported that the drug resistant state was not reversible. While drug removal still induced increases in pERK, there was no loss of fitness, but instead induction of EMT and chemoresistance [35]. This study highlights the potential for intermittent dosing in a separate cancer type, albeit only with specific mechanisms of resistance.

A study by Amin et al. in ALK^+^ NSCLC found that copy number gains in the ALK fusion oncogene could drive resistance and addiction to ALK inhibitors. They observed dependence on continued culture in ALK inhibitors, highlighting another instance in which intermittent dosing may prolong onset of resistance. They did not, however, probe the role of ERK or other downstream ALK effectors in this phenotype [164]. A 2017 study by Ceccon and colleagues found similar results in NPM-ALK fusion Anaplastic Large Cell Lymphoma (ALCL) models [165]. They explored the biological rationale for the sequestering and inactivation of approximately half of the fusion chimeras in ALCL models, and found that this buffering was necessary for cell survival. Excessive ALK signaling, driven by overexpression of ALK or inhibition of nuclear import, resulted in proliferative arrest, apoptosis, and activation of the ATM-Chk2 DNA-damage response pathway, all of which were rescued through inhibition of ERK/MAPK signaling. This vulnerability also manifested as drug addiction in ALCL cells resistant to ALK inhibitors through ALK amplifications, where drug discontinuation led to apoptosis and DNA damage. Long term withdrawal in drug addicted cell restored sensitivity to ALK inhibitors, again highlighting the potential for intermittent dosing to forestall drug resistance [165].

In models of gastric cancer, Funakoshi and colleagues observed resistance and addiction to MET inhibitors mediated by MET amplification [166]. In drug resistant cells, drug withdrawal led to cell cycle arrest and DNA damage, which could be rescued by partial MET inhibition. The authors noted increased STAT3 phosphorylation after drug removal, however did not further characterize STAT signaling or other pathways downstream of MET.

### Pharmacological induction of ERK hyperactivation

Data indicating that cancer cells rely on optimal levels of signaling through ERK and other oncogenic pathways continues to accumulate. Pharmacological efforts to exploit this vulnerability have yielded compounds inhibiting targets identified from preclinical work, as well as agonists that can directly activate ERK signaling.

#### DUSP6 inhibitors

BCI was one of the first studied small molecule inhibitors that activate the ERK/MAPK pathway, identified by Molina and colleagues in 2009 through a transgenic zebrafish chemical screen for compounds that modulate fibroblast growth factor signaling [167]. Using docking simulations and in vitro studies, BCI was found to inhibit the catalytic activation of DUSP6 induced by ERK2 substrate binding [167], a mechanism later validated by others [168].

Shojaee and colleagues investigated the potential of inhibition of DUSP6 in acute lymphoblastic leukemia (ALL) [169]. In pre-B cells, they found that overexpression of common oncogenes (BRC-ABL1, NRAS^G12D^) induced cell death, suggesting transformed cells are dependent on additional factors to survive. They found that transformation in ALL models was dependent on negative regulation of ERK signaling through factors such as DUSP6 and SPRY2. Separate ALL models were sensitive to BCI, which was found to induce ERK hyperactivation and p53-dependent cell death. Cells made resistant to common ALL therapies were also found to be sensitive to BCI, suggesting use of hyperactivation to overcome the drug resistant state [169].

Unni et al. saw similar results following exposure of lung cancer cell lines to BCI. Their results showed that inhibition of DUSP6 by BCI was more toxic to cells bearing a *KRAS* or *EGFR* mutations. BCI toxicity was also dependent on ERK signaling, indicating a reliance on ERK activity for sensitivity to DUSP6 inhibition [32]. Wu and colleagues tested BCI in models of gastric cancer. They also found that BCI treatment induces higher levels of ERK signaling and p53 mediated cell death. It was found to enhance the effects of cisplatin [170], suggesting synergy between ERK hyperactivation and more traditional therapy.

A study by Ramkissoon and colleagues explored the knockdown of DUSP6 in malignant peripheral nerve sheath tumors, where elevated MAPK signaling plays a role in tumorigenesis [171]. Knockdown of DUSP6 resulted in decreased MPNST growth and hyperactivation of both ERK and JNK [171]. Further in vivo tests treating patient-derived and cell-line xenografts of MPNST showed BCI induced tumor fibrosis, necrosis and reduced tumor volume [171]. Martin-Vega and colleagues aimed to investigate the link between ERK signaling and ASCL1 expression, a SCLC subtype marker. It was previously shown that ERK activity is negatively correlated with ASCL1 expression [114, 115]. They identified the phosphatase DUSP6 as a key negative regulator of ERK signaling expressed in ASLC1 positive SCLC. Pharmacological inhibition of DUSP6 with BCI resulted in increased ERK signaling and decreased cell proliferation specifically in this subtype, and had no effect in the POU2F3 or NEUROD1 SCLC subtypes [172].

#### Inhibitors targeting other phosphatases

In addition to small-molecule inhibitors of phosphatases that act on ERK, there are inhibitors of phosphatases acting on other parts of the MAPK signaling pathway. One such inhibitor resulting in hyperactivation of oncogenic signaling is NSC 95,397, a quinone-based small molecule [173]. NSC 95,397 is an inhibitor of dual-specificity phosphatases, including DUSP1 (MKP-1), a MAPK phosphatase targeting JNK, p38 and ERK [173, 174]. From tests with three colon cancer cell lines, NSC 95,397 was shown to inhibit cancer cell proliferation and induce apoptosis [173]. They observed pERK following inhibition of MKP-1, however did not rule out other mediators of toxicity [173].

Dias et al. investigated LB-100, an inhibitor of protein phosphatase 2 A (PP2A), and extensively characterized its mechanism in models of colorectal cancer. PP2A is a serine/threonine phosphatase that acts on several cancer related pathways, including ERK, AKT and STAT5 [175]. They first demonstrate that LB-100 induces mitogenic signaling and cell stress responses in treated cells. Through a CRISPR knockout and CRISPR activation screen, they confirmed that ERK/MAPK signaling and Wnt/β-catenin signaling was responsible for mediating toxicity LB-100 toxicity. They next performed a drug screen and uncovered that WEE1 inhibition with adavosertib is synthetic lethal with LB-100. WEE1 is an important regulator of cell cycle progression [176], and the combination was found to induce cell death through mitotic catastrophe following DNA replication stress. Adavosertib and LB-100 were synergistic across models of in vitro CRC, PDAC and cholangiocarcinoma, and in PDX models. They also profiled mechanisms of resistance to the combination. While resistant cells do emerge, these tend to have decreased ERK and Wnt signaling, display poorer anchorage-independent growth ability, and have decreased aneuploidy, suggesting resistant cells are less malignant than the parental ones. In a separate study, the same group identify that LB-100 induces neoantigens by impairing RNA splicing [177], suggesting potential synergy with immunotherapy.

#### Other small molecule inducers of pERK

Studies have identified several drugs that induce pERK by acting as MAPK pathway agonists, as opposed to inhibiting negative regulators. In 2017, a study by Satoh and colleagues identified a 1’-acetoxychavicol acetate analogue compound; ACA-28, which they showed was able to impair growth and induce apoptosis in both melanoma cells and transformed NIH/3T3 cells. The authors partially rescued proliferative arrest by treating with a MEK inhibitor, suggesting ERK involvement. However, they did not identify a mechanism of action of ACA-28 [178]. In later work, ACA-28 was found to partially mediate cell death through induction of ROS [179], a cell stressor previously shown to inhibit phosphatases acting on the ERK/MAPK pathway [180, 181].

Phorbol 12-myristate 13-acetate (PMA), an activator of PKC has also been shown to induce ERK hyperactivation through a mechanism involving ERK catalytic activity and effectors downstream of the MAPK pathway [120]. Tests in the HMCB cell line showed that PMA activated MAPK signaling, as measured using ERK1/2 substrates and ERK1/2-regulated proteins [120]. Cell proliferation was impaired upon PMA treatment, and dox induced ERK2 overexpression only furthered this effect [120]. Elaiophylin, an autophagy inhibitor, was found to induce ERK hyperactivation in ovarian cancer cells [182]. GSEA indicated enriched MAPK signaling in SKOV3 cells treated with elaiophylin. Elevated MAPK signaling was linked to elaiophylin-induced ER stress and paraptosis in ovarian cancer cells [182]. Elaiophylin preferentially killed drug-resistant ovarian cancer cells bearing elevated MAPK levels, suggesting a use case in treatment refractory cancers [182].

#### MAPK pathway agonists

Xu and colleagues aimed to identify molecules to target KRAS. They screened a library of ~ 300,000 compounds from the National Cancer Institute against the GTP/GDP pocket of KRAS [183]. They identified KRA-533 as the compound with the strongest effect against human LC cell lines. KRA-533 treatment induced accumulation of the active GTP-bound state of KRAS, suggesting it was acting as a KRAS agonist. This effect was stronger in cells with mutant KRAS than wild-type KRAS. In lung cancer cell lines, KRA-533 induced apoptosis and autophagic cell death. While the authors noted increased ERK signaling, they did not rule out the involvement of other pathways in the phenotype. In xenograft and genetically engineered KRAS mutant lung cancer mouse models, the authors observe impaired tumor growth and a favorable safety profile [183].

### Clinical trials relating to ERK hyperactivation

**Fig. 2:**
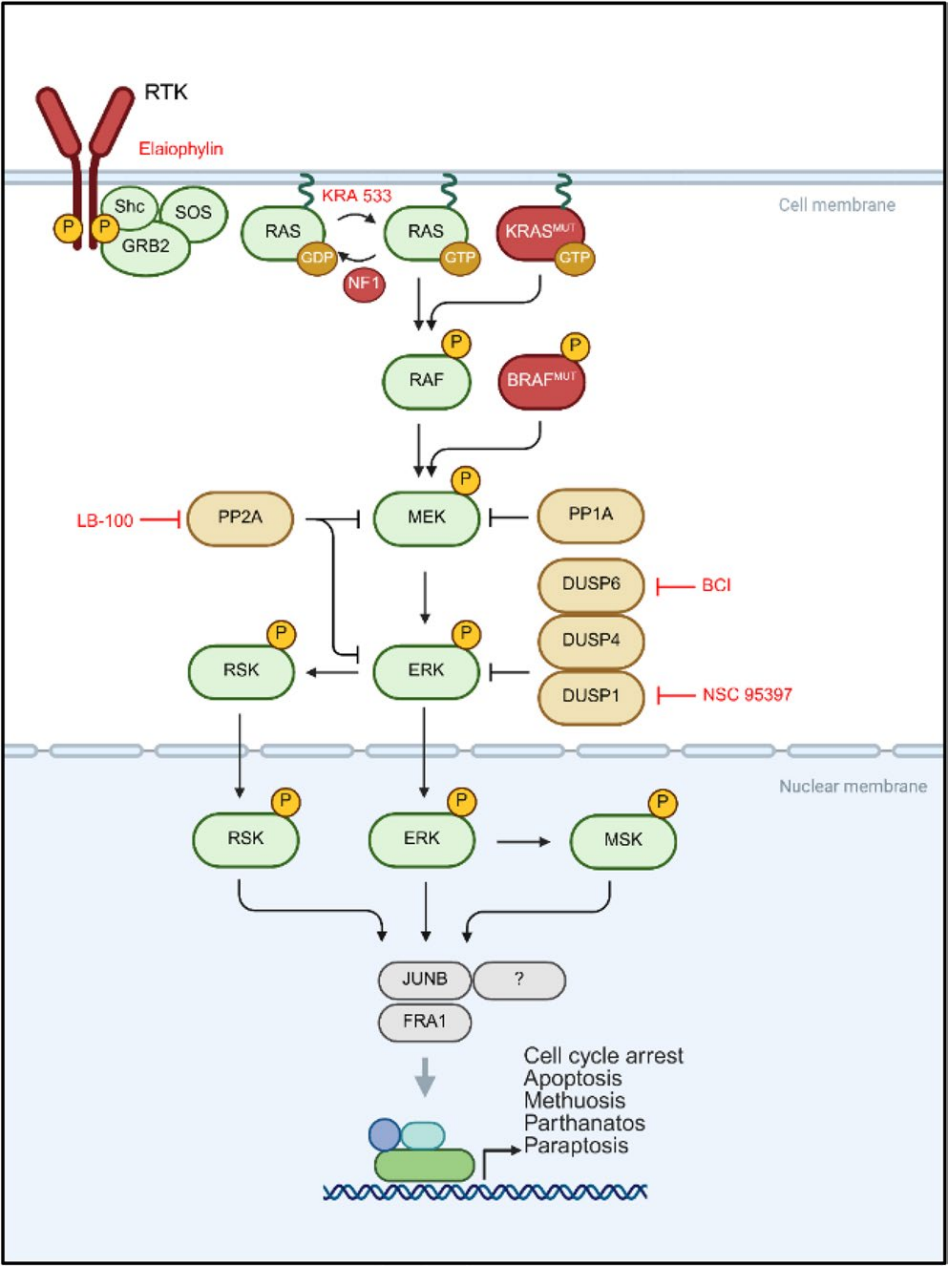
Negative regulators of ERK/MAPK signaling and small molecules inducers of ERK hyperactivation. Figure made with BioRender

Extensive modelling of the ERK hyperactivation phenotype across several cancer types with multiple model systems has defined important pathways and individual proteins regulating ERK induced toxicity ERK (Fig. 2, Table 2), some of which have informed clinical trials (Table 3). Case reports of melanoma patient tumors regressing following drug removal, as well as subsequent in vitro investigation of these results, have inspired clinical trials aimed at harnessing BRAF amplification and subsequent ERK hyperactivation. A phase II trial conducted in Belgium observed successful rechallenge of melanoma patients after they progressed on BRAF inhibitors [185]. Two later phase II trials found no benefit in intermittent dosing, and that it decreased progression-free survival in enrolled patients [186, 187]. Another clinical trial evaluating the use of vorinostat administered following progression on BRAF and MEK inhibitors to target drug resistant cells only observed a 9% response rate [162]. Successful implementation of intermittent dosing will require careful assessment of mechanisms of resistance in patients prior to drug discontinuation, and may require strategies to potentiate ERK induced lethality. Additional biomarkers may be necessary if additional therapeutics are to be leveraged to potentiate the effects of drug discontinuation and ERK hyperactivation. LB-100 has advanced to clinical trials. It was found to increase sensitivity to chemotherapy [189], and was reported to have a favorable safety profile in a phase 1 trial [188]. A phase Ib trial is currently underway evaluating the combination of LB-100 with carboplatin, etoposide, and atezolizumab for the treatment of SCLC. To date, no other individual compound or combined treatment strategy aiming to treat cancers by hyperactivating ERK or other oncogenic pathways have progressed to the clinic.

**Table 2:** Models used and observed outcomes from studies reporting ERK hyperactivation mediated toxicity in cancer cells

Model	Hyperactivation mechanism	Outcome	Ref.
H209, H510	ΔRaf-1:ER	Cell cycle arrest, p27^Kip1^ upregulation	[102]
DMS53	ΔRaf-1:ER	Cell cycle arrest, p16^INK4A^ upregulation	[103]
LNCaP	ΔRaf-1:ER	Cell cycle arrest, p21^Cip1^ upregulation	[104, 109]
Normal human astrocytes	ΔRaf-1:ER	Irreversible growth arrest, upregulation of SA-ßGal and p16^INK4A^ expression	[107]
U87, U251, and SF126	ΔRaf-1:ER	Reversible growth arrest associated with increased p21^Cip1^ expression	
TT	ΔRaf-1:ER	Cell cycle arrest, LIF/JAK/STAT3 autocrine/paracrine signaling	[108]
Huh-7D12, HepG2, MCF-7	pCMV-MEK1^S218D/S222D^, MEK2^S222D/S226D^	Cell cycle arrest, p21^Cip1^ upregulation	[111]
LNCAP, U251	ΔRaf-1:ER + pHAGE-ERK1, pHAGE-ERK2	Caspase dependent apoptosis	[112]
H510, H720, H69, DMS79	pInducer20 – MEK1^S217D/S221D^	Cell cycle arrest, senescence	[114]
LNCaP	pBluescript SK – ERK2^L73P/S151D^	Cell cycle arrest, upregulation of p16^INK4A^ and p2^Cip1^	[116]
A375	ERK1/ERK2 mutants	Proliferation arrest	[118, 119]
A375	pXP1509A-ERK1^WT^ pXP1509A-ERK2^WT^	Caspase independent cell death	[120]
HMCB	PMA	Proliferation arrest	
HeLa	DUSP3 KO	Cell cycle arrest, upregulation of p21^Cip1^	[124]
LNCaP, SKMEL1, SKMEL28	shMortalin	Proliferation arrest, p21^Cip1^ upregulation, apoptosis,	[117, 125, 126]
SKMEL28, SKMEL24, COLO829, RVH-421	DUSP4 KD	Proliferation arrest, apoptosis	[131]
PC9, H358, H1975	pInducer20-KRAS^G12V^ pInducer20-EGFR^L858R^	Proliferation arrest, apoptosis, methuosis	[32, 139]
A549, H460, H23, H358, H2122, H1650, H2009, H2030, PC9, H1975	BCI, DUSP6 KD	Proliferation arrest	[32]
C57BL/6	Kras*; TetO-EGFR^L858R^; Rosa26-LSLrtTAKIKI Kras-SL612Vgeo; TetO-EGFR^L858R^; Rosa26-LSLrtTAKIKI	Proliferation arrest, apoptosis	[143]
C57BL/6	Braf^CA/+^ Kras2^LSL/+^	Proliferation arrest, upregulation of p16^INK4A^, p15^INK4B^, SA-ßGal	[144]
Melanoma clone 21	pTet-NRAS^Q61R^	Proliferation arrest, upregulation of p21^Cip1^, SA-ßGal	[145]
IGR1, A375	sgDUSP4, sgPPP2R2A	Proliferation arrest	[146]
A375, SK-MEL-28	shDUSP4	Proliferation arrest	[148]
501mel	shPPP6C	Proliferation arrest	[149]
WM2664, MALME3M, RVH421, HS294T, A375, HT144	sgDUSP4, sgDUSP6	Proliferation arrest	[31]
BRAFi resistant melanoma PDX	BRAF Amplification, drug withdrawal (BRAFi)	Tumor shrinkage	[33]
A375	pLX304-EGFR	Proliferation arrest, upregulation of p21^Cip1^, p27 ^Kip1^	[158]
M249, M397, M395, M229, SKMEL28	Drug withdrawal, (BRAFi, MEKi)	Proliferation arrest	[159]
M397, M395, M238, M229, SKMEL28	Drug withdrawal (BRAFi, MEKi)	Cell cycle slowdown	[160]
M249, M245, M207	Drug withdrawal (BRAFi, MEKi)	AIF-dependent, caspase-3–independent cell death	
451Lu, A375, A101D, Mel888	Drug withdrawal (BRAFi, MEKi)	Cell death	[34]
HCC827	Drug withdrawal (EGFRi)	Proliferation arrest, apoptosis	[163]
H358*sgRB1*#4, H358, H23, H1792	Drug withdrawal (MEKi) pInducer20-KRAS^G12C^	Proliferation arrest, apoptosis	[36]
C6244, HT6244	Drug withdrawal (MEKi)	Cell cycle arrest, p57^Kip2^ upregulation, caspase dependent cell death	[35]
SU-DHL-1, COST, TS, KARPAS 299	pCCL-NPM-ALK, drug withdrawal (ALKi)	Proliferation arrest, apoptosis	[165]
Patient-derived ALL cell lines	BCI	p53-dependent cell death, tumor shrinking	[169]
SGC7901, BGC823	BCI	Proliferative arrest, apoptosis, tumor shrinkage	[170]
iHSC-1γ, ST8814, S462.TY, STS26T	BCI	Proliferation arrest, tumor shrinking	[171]
HCC1833	BCI, PMA	Cell cycle slowdown, apoptosis	[172]
SW480, SW620, DLD-1	NSC 95,397	Proliferation arrest, p21^Cip1^ upregulation apoptosis	[173]
DiFi, DLD-1, HCT-116, HT-29, LoVo, RKO, SW-480	LB-100	Proliferation arrest	[177, 184]
SK-MEL-28, A4-15, NIH/3T3	ACA-28	Proliferation arrest, apoptosis	[178]
SKOV3, OVCAR8, UWB1.289, SW626	Elaiophylin	Proliferation arrest, paraptosis	[182]
A549, C57BL-6	KRA-533	Proliferation arrest, apoptosis, tumor shrinking	[183]

**Table 3:** Clinical trials investigating ERK hyperactivation

Clinical trial registration	Phase	Cancer type	Treatment	Ref.
NCT02296996	Phase 2	Advanced unresectable BRAF^V600E/K^ melanoma	Dabrafenitb + trametinib	[185]
NCT02196181	Phase 2	Advanced unresectable BRAF^V600E/K^ melanoma	Dabrafenitb + trametinib	[186]
NCT02583516	Phase 2	Advanced unresectable BRAF^V600E/K^ melanoma	Vemurafenib + Cobimetinib	[187]
NCT02836548	Phase 1/2	Advanced unresectable BRAF^V600E/K^ melanoma	Vorinostat	[162]
NCT01837667	Phase 1	Progressive or metastatic solid tumors	LB-100	[188]
NCT04560972	Phase 1	Extensive stage small cell lung carcinoma	LB100 + carboplatin/etoposide/atezolizumab	N/A

#### Remaining questions and future directions

**Fig. 3:**
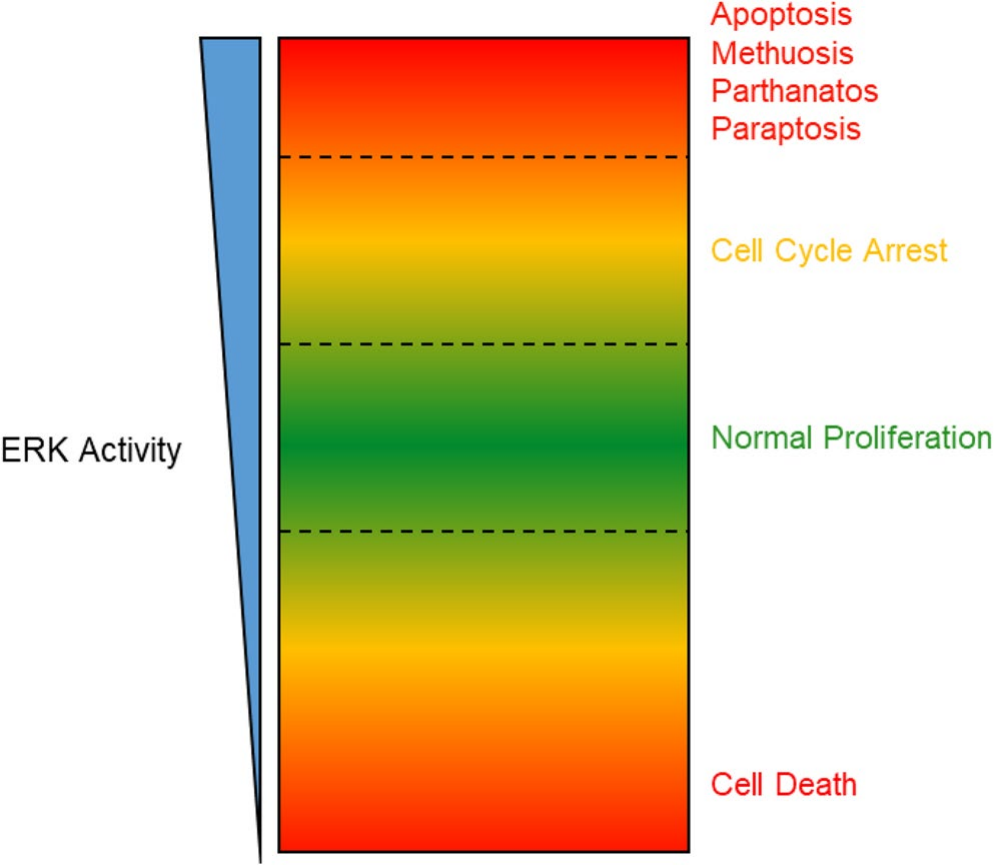
ERK pathway signaling intensity in cancer cells and associated outcomes

ERK hyperactivation remains largely an umbrella term to describe a phenotype of proliferative arrest induced by high levels of ERK signaling that is rescued by ERK inhibition. This loose definition is often accompanied by other shared features, including cell cycle arrest, senescence, apoptosis and caspase-dependent/independent cell death (Fig. 3). Many studies to date have focused on reporting ERK hyperactivation in new contexts, highlighting that this vulnerability exists across multiple cancer types. The severity and mechanism appear tissue and context-dependent, underscoring the need for further characterization in a variety of models. A better understating of ERK signaling dynamics and balance within cells, as well as the feedback mechanisms, will be important in predicting outcomes and refining future clinical strategies for maximum effect.

#### ERK toxicity mediators and cellular outcomes

While pre-clinical research identified multiple alterations capable of inducing ERK hyperactivation, the dynamics of ERK signaling, as well as mediators of toxicity downstream of ERK, remain unclear. In models of drug addiction, our group and others found that lethality was associated with a pERK increase that was sustained past 3–5 days [35, 36]. Studies have identified thresholds of ERK activation necessary for lethality; however, have not examined how ERK spike duration affects toxicity. Regarding downstream mediators, functional genetic screens performed in models of hyperactivation have identified ERK2, kinases that activate ERK signaling, or TFs downstream of ERK, as mediators of toxicity [32, 34, 120]. Transcriptome profiling has revealed upregulation of stress response signaling pathways including p38 and unfolded protein response [120, 190]. This data has reinforced the importance of ERK signaling in mediating lethality in these models, however, has not revealed the trigger for proliferative arrest. Efforts to potentiate ERK toxicity have provided some insight towards the mediators of toxicity. Dias and colleagues found that WEE1 inhibition was synthetic lethal with oncogenic hyperactivation induced by pharmacological inhibition of PP2A, suggesting replicative stress and dependence on G2/M checkpoint integrity [184]. Another study found that DNA damaging agents potentiated ERK mediated lethality, again suggesting a dependence on DNA replication [160]. Work from our group and others has found that ERK hyperactivation induces proteotoxic stress [36, 120]. A study also found that the drug addicted state of BRAF resistant melanoma is sensitive to induction of ROS [161]. To date, no shared mechanism or vulnerability underlying ERK hyperactivation has been found. While multiple cancer types appear to be sensitive to ERK mediated toxicity, the mechanisms of proliferative arrest are likely tissue and level specific. A better understating of outcomes and compensatory mechanisms induced by ERK hyperactivation may prove necessary to develop strategies to potentiate responses.

With regards to the cellular consequences of ERK hyperactivation, diverse responses are elicited, including cell death, senescence, and cell cycle arrest; multiple phenotypes can be observed if certain thresholds of ERK are surpassed [112]. The extent of p53’s involvement in senescence, cell cycle arrest and death in the context of ERK hyperactivation remains unclear, as both p53-dependent and independent responses have been observed [125]. Many groups have reported proliferation arrest mediated through senescence, mirroring the OIS phenotype of non-transformed cells. The induction of apoptosis resulting from ERK hyperactivation appear to run contrary to ERK’s defined role in promoting survival, but are supported by extensive reports highlighting ERK’s role in mediating response to cell stressors, including many compounds used for cancer treatment [27]. The induction of alternate mechanisms of cell death, including methuosis, paraptosis and parthanatos, highlight our incomplete understanding of ERK signaling in cancer cells.

#### Parsing the functional specificity of ERK isoforms

The role of specific ERK isoforms in mediating ERK toxicity remains unclear. Work on cancer initiation indicates functional redundancy between ERK1 and ERK2 [191]. In the context of ERK mediated lethality, several studies highlight the functional differences between ERK1/2, while others point to them being interchangeable in a cell-type specific manner. ERK2 has been implicated by CRISPR screening as a mediator of toxicity in LUAD and the proliferative arrest associated with the drug addiction phenotype in melanoma [32–34]. Additionally, ERK1^R84H^, an intrinsically active ERK1 variant, has been implicated in hepatocellular carcinoma development in mice [122]. Studies citing a degree of functional redundancy report no significant changes in ERK signaling targets expression level with knockdown of either isoform [111, 192].

It is important to note that although both ERK1 and 2 are ubiquitously expressed, ERK2 is more highly expressed in most mammals, leading the question of whether the observed phenotypes attributed to overexpression or knockdown of ERK isoforms are instead a result of the global ERK expression level [193]. In many cancer models, the prominence of ERK2 in proliferation has been questioned, as studies in mouse fibroblasts suggest that ERK2’s impact on proliferation can be attributed to its higher expression level [194, 195]. This indicates that, alongside the notion of ERK1/2 specific roles during disease and development, the role and regulation of total ERK levels should be considered in future research.

#### Interplay between ERK hyperactivation and immune response

The majority of ERK hyperactivation studies are conducted in vitro, omitting potential effects from immune involvement. In a mouse model, Ciwoski and colleagues demonstrated that increased MAPK signaling by expressing both oncogenes BRAF and NRAS in melanoma them more susceptible to detection and cell killing by immune cells [145]. Dias and colleagues reported generation of neo-antigens following ERK hyperactivation induced by LB-100 [177]. Additionally, hyperactive MAPK signaling has been shown to have immunomodulatory effects during tumorigenesis and chronic disease on various levels [196, 197]. Synergy of ERK hyperactivation with immunotherapy is an avenue that invites further exploration.

#### Balancing therapeutic efficacy and oncogenic risk in ERK hyperactivation

ERK hyperactivation is a double-edged sword as it holds therapeutic potential that would be broadly applicable to MAPK-driven cancers but is also capable of accelerating tumor progression in certain contexts. The toxic phenotype of ERK hyperactivation is varied and does not necessarily result in cell death, which presents a potential hurdle to clinical translation. As such, the therapeutic window of ERK levels should be carefully assessed to maximize therapeutic benefit while avoiding induction of uncontrolled proliferation. An important consideration in leveraging ERK hyperactivation is the effect on normal cells if therapies are delivered systemically. Trials with BRAF inhibitors in melanoma reported secondary cancers emerging [198, 199] due to their ability to paradoxically activate ERK signaling, promoting transformation in cells with pre-existing MAPK activating mutations [133, 200–202]. For melanoma patients, this has been remedied by co-treatment with a MEK inhibitor [22]. For the study of ERK hyperactivation, this work highlights the potential consequences of continuous stimulation of ERK/MAPK signaling. While we cannot predict the effect of future therapies, these findings highlight the importance that any potential activators of MAPK signaling should be either tumor specific, follow a dosing schedule that limits transformation of normal cells, or be cycled intermittently with currently approved treatments as a means to delay resistance.

## Conclusions

Overall, targeting cancer cells through ERK hyperactivation holds promise across multiple tumor types, especially when paired with currently approved therapeutics. More work is required to develop inhibitors to targets identified in pre-clinical studies and to define the therapeutic window .

## Data Availability

No datasets were generated for this manuscript. The results shown in Table 1 are in whole based upon data generated by the TCGA Research Network: https://www.cancer.gov/tcga."
